# Duration of late-follicular elevated progesterone and *in vitro* fertilization outcomes in pituitary down-regulation treatment cycles

**DOI:** 10.3389/fendo.2023.1186146

**Published:** 2023-06-19

**Authors:** Jiaxin Zhang, Xiaofei Ge, Zhiqin Bu

**Affiliations:** Reproductive Medical Center, Henan Province Key Laboratory for Reproduction and Genetics, The First Affiliated Hospital of Zhengzhou University, Zhengzhou, China

**Keywords:** late-follicular elevated progesterone, duration, clinical pregnancy, *in vitro* fertilization, infertility

## Abstract

**Background:**

The objective of this study was to explore whether the duration of LFEP (late-follicular elevated progesterone) affected pregnancy outcomes in IVF (*in vitro* fertilization) patients treated with pituitary downregulation protocols.

**Method:**

Patients with their first IVF/ICSI cycles between January 2016 and December 2016 were included. LFEP was set either at P > 1.0ng/ml or P > 1.5ng/ml. Clinical pregnancy rate was compared among three different groups (no LFEP; LFEP for 1 day; LFEP for ≥ 2 days). Then multivariate logistic regression analysis was performed to explore the influencing factors of clinical pregnancy rate.

**Results:**

This retrospective analysis involved 3,521 first IVF/ICSI cycles with fresh embryo transfers. Clinical pregnancy rate was the lowest in patients with a LFEP duration of ≥ 2 days, irrespective of whether LFEP was defined as P > 1.0 ng/ml (68.79% vs. 63.02% vs. 56.20%; *P* = 0.000) or as P > 1.5 ng/ml (67.24% vs. 55.95% vs. 45.51%; *P* = 0.000). In addition, LFEP duration was significantly associated with clinical pregnancy outcomes in unadjusted logistic regression analysis. However, in multivariate regression models after adjusting confounders, adjusted OR for LFEP duration (≥ 2 days) in the two models was 0.808 (*P* = 0.064; LFEP as P > 1.0 ng/ml) and 0.720 (*P* = 0.098; LFEP as P > 1.5 ng/ml), respectively.

**Conclusion:**

LFEP adversely affects clinical pregnancy outcomes. However, the duration of LFEP seems to have no influence on the clinical pregnancy rate in pituitary downregulation treatment cycles.

## Introduction

The influence of late-follicular elevated progesterone (LFEP) on *in vitro* fertilization outcomes has been controversial for decades. It was reported that the subtle progesterone rise on the day of human chorionic gonadotrophin (hCG) triggering can reach 5%–71% (1, 2). Originally researchers mistakenly regarded this phenomenon as premature luteinization, while the rise in progesterone (P) was not always accompanied by an increase in luteinizing hormone (LH). Up to now, the exact cause of P elevation in the follicular phase of ovarian stimulation has not been fully elucidated.

Many clinical studies have examined the effects of a subtle P rise on the day of hCG triggering on pregnancy outcomes (3–5). A meta-analysis with more than 60,000 cycles supposed that elevated P on the day of hCG administration is associated with a decreased probability of pregnancy achievement in fresh ET cycles in women undergoing ovarian stimulation using GnRH analogues and gonadotrophin (6). The adverse effect of P elevation was only present in fresh IVF cycles rather than in frozen-thawed or donor/recipient cycles, and the threshold of P ranged from 0.8-1.9 ng/ml (6). In addition, many other elevated P-related indicators were studied to predict pregnancy outcomes, including the P-to-estradiol ratio (P/E_2_), P-to-oocyte, P-to-follicle, and P-to-mature oocyte index (PMOI) (7–10). However, these indexes failed to perform superiorly in pregnancy rate.

In 2012, Huang et al. performed an interesting study on the duration of LFEP for the first time. It seemed that the duration of P elevation played a major role in pregnancy outcomes (11). The cumulative P exposure was more in line with how P regulated endometrial development. However, a more recent study suggested that there was a lack of benefit in measuring serum P in the days preceding hCG administration, since the live birth rate (LBR) in women with LFEP 0, 1, and > 1 day did not vary significantly from those with LFEP detected only on the day of hCG administration (12).

In these two previous studies, the criterion for LFEP was P > 1.0 ng/ml on the day of hCG administration. Patients were treated with either multiple ovarian stimulation protocols or GnRH-ant protocol. In the current study with a larger population, we explored the impact of LFEP duration on IVF outcomes in women treated with pituitary downregulation treatment cycles under two different conditions: LFEP was defined as P > 1.0 ng/ml or as P > 1.5 ng/ml.

## Materials and methods

This study was approved by the Institutional Review Board (IRB) of the First Affiliated Hospital of Zhengzhou University. Written informed consent was obtained from all patients before IVF treatment for physicians collecting basic information and treatment data. Data in this study were from the Clinical Reproductive Medicine Management System/Electronic Medical Record Cohort Database (CCRM/EMRCD) from the Reproductive Medical Center, First Affiliated Hospital of Zhengzhou University.

### Study population

Infertile women undergoing their first IVF/ICSI (intracytoplasmic sperm injection) cycle treatment with a GnRH agonist pituitary suppression protocol from January 2016 to December 2016 were enrolled in this retrospective study. All patients underwent fresh embryo transfers. The exclusion criteria were as follows (1): uterine malformation (2); oocyte-donation cycles; (3) recurrent spontaneous abortion and repeated implantation failure; and (4) pre-implantation genetic testing cycles.

### Assisted reproductive technology procedures

Pituitary downregulation and controlled ovarian stimulation were performed as described in a previous study (13). Of relevance, pituitary suppression using a GnRH agonist was achieved with Diphereline (GnRH-a, Ferring, Germany) when follicle-stimulating hormone (FSH) ≤ 5 IU/L, E_2_ ≤ 50 pg/ml, LH ≤ 5 mIU/mL, and endometrial thickness ≤5 mm. During ovarian stimulation, LH, E_2_, and P levels were collected when the diameter of the largest follicle reached 14 mm. Ovulation triggering criteria were: the diameter of the leading follicle was more than 20 mm; at least three follicles > 17 mm. Then, 36-38 hours after hCG trigging, oocytes were retrieved. The patients underwent the standardized procedures of the fertility center; most took a P test in the morning, and the frequency of the P test was every day. Embryo transfer was performed on day 3 or day 5 after fertilization. All the patients had two cleavage embryos or one blastocyst embryo transplanted. Clinical pregnancy was defined by the presence of a fetal heart beat 35 days after the day of embryo transfer. Early spontaneous abortion in this article was defined as spontaneous pregnancy loss after sonographic visualization of an intrauterine gestational sac before 12 weeks of gestation.

### Statistical analysis

Firstly, basic characteristics and pregnancy outcomes were compared between patients from different LFEP duration groups (LFEP was defined as P > 1.0ng/ml or P > 1.5ng/ml). The patients were divided according to the duration of LFEP (0 day, 1 day, and ≥ 2 days) on the day of hCG triggering. Then, logistic regression analysis was used to explore the impact of LFEP duration and IVF outcomes.

Statistical analysis was performed with SPSS 24.0 software. Continuous data were presented as median ± standard deviation (M ± SD) and were analyzed using Student’s t-test; three groups were analyzed by ANOVA analysis. Categorical data was described by the number of cases and percentages, and analyzed using Pearson’s chi-squared test. *P*-value was considered significant whenever < 0.05 in general situations.

## Results

A cohort of 3,521 first IVF/ICSI cycles with fresh embryo transfers were included in the study. The age of the participants was between 20-48 years old, with a median ± standard deviation of age of 30.5 ± 4.69. Basic demographic parameters were different between the different LFEP duration groups (**Table 1**). When the LFEP was defined as P > 1.0ng/ml, there were 2,041 patients in the 0 day group, 795 patients in the 1 day group, and 685 patients in the ≥ 2 days group. Clinical pregnancy rate was significantly lower (68.79% vs. 63.02% vs. 56.20%, respectively; *P* < 0.001) and spontaneous abortion rate was higher (8.55% vs. 8.38% vs. 10.39%, respectively; *P* < 0.001) in the ≥ 2 days group as compared with those in the 0 day and 1 day groups.

**Table 1:** Demographic and pregnancy outcomes according to the duration of LFEP>1.00 ng/ml (n=3521).

	Duration of LFEP > 1.00 ng/mL			p-value
	0 day(n=2041)	1 day (n=795)	≥ 2 days (n=685)	
**Age (y)**	30.32 ± 4.65^a^	30.55 ± 4.75^b^	31.16 ± 4.73^ab^	<0.001
**Duration of infertility (y)**	3.99 ± 2.92^a^	3.97 ± 2.91^b^	4.36 ± 3.21^ab^	0.017
**BMI (Kg/m^2^)**	22.99 ± 3.21^a^	22.49 ± 3.10^a^	22.33 ± 2.84^a^	<0.001
**Basic FSH (mIU/mL)**	6.35 ± 1.84	6.28 ± 1.71	6.34 ± 1.69	0.679
**Gn duration (days)**	13.88 ± 2.20^a^	14.03 ± 1.94^b^	14.28 ± 1.92^ab^	<0.001
**Gn dosage (IU)**	2569.23 ± 1003.91^a^	2598.49 ± 907.47^b^	2826.65 ± 834.58^ab^	<0.001
**Oocytes retrieved**	12.40 ± 5.07^a^	14.13 ± 5.24^a^	14.43 ± 5.41^a^	<0.001
**No. embryos transferred**	1.90 ± 0.31^ab^	1.85 ± 0.36^a^	1.83 ± 0.37^b^	<0.001
**Fertilization methods**				0.037
**IVF**	1540(75.45%)	622(78.24%)	547(79.86%)	
**ICSI**	501(24.55%)	173(21.76%)	138(20.14%)	
**HCG day hormone levels**				
**LH (mIU/mL)**	1.06 ± 1.22^a^	1.29 ± 1.50^ab^	1.01 ± 1.18^b^	<0.001
**E_2_ (pg/ml)**	2840.50 ± 1354.88^a^	3818.75 ± 1679.91^a^	4166.71 ± 1781.72^a^	<0.001
**P (ng/ml)**	0.59 ± 0.24^a^	1.31 ± 0.28^a^	1.76 ± 0.48^a^	<0.001
**Clinical pregnancy rate**	1404(68.79%)^a^	501(63.02%)^a^	385(56.20%)^a^	<0.001
**Abortion rate**	120(8.55%)^a^	42(8.38%)^b^	40(10.39%)^ab^	<0.001

a, b Post-hoc pairwise comparisons P-value <0.05 for values with the same superscript letter; continuous variables are presented as mean ± SD; LFEP, late follicular elevated progesterone; BMI, body mass index; FSH, follicle-stimulating hormone; Gn, gonadotropin; ICSI, intracytoplasmic sperm injection; HCG, human chorionic gonadotropin; LH, luteinizing hormone; E_2_, estradiol; P, progesterone.

When the LFEP was defined as P >1.5 ng/ml, there were 2,934 patients in the 0 day group, 420 patients in the 1 day group, and 127 patients in the ≥ 2 days group, as shown in **Table 2**. The differences between three groups were similar to those when LFEP was defined as P >1.0 ng/ml. The clinical pregnancy rates in the three groups were 67.24%, 55.95%, and 45.51%, respectively. The differences reached statistical significance. However, the spontaneous abortion rate was comparable between the three groups (8.89% vs. 9.79% vs. 9.21%, respectively, *P* < 0.001).

**Table 2:** Demographic and pregnancy outcomes according to the duration of LFEP>1.50 ng/ml (n=3521).

	Duration of LFEP > 1.50 ng/mL			p-value
	0 day (n=2934)	1 day (n=420)	≥ 2 days (n=167)	
**Age (y)**	30.39 ± 4.66^a^	31.30 ± 4.81^b^	31.26 ± 4.75^ab^	<0.001
**Duration of infertility (y)**	4.02 ± 2.95	4.28 ± 3.21	4.20 ± 2.93	0.234
**BMI (Kg/m^2^)**	22.84 ± 3.19^a^	22.42 ± 2.85^b^	21.99 ± 2.65^ab^	<0.001
**Basic FSH (mIU/mL)**	6.33 ± 1.79	6.32 ± 1.70	6.36 ± 1.70	0.968
**Gn duration (days)**	13.95 ± 2.13^a^	14.03 ± 1.83	14.53 ± 1.95^a^	0.002
**Gn dosage (IU)**	2594.76 ± 971.22^a^	2733.05 ± 864.07^a^	2903.96 ± 853.71^a^	<0.001
**Oocytes retrieved**	12.86 ± 5.18^ab^	14.57 ± 5.20^a^	15.40 ± 5.69^b^	<0.001
**No. embryos transferred**	1.88 ± 0.32^ab^	1.83 ± 0.37^a^	1.83 ± 0.38^b^	0.003
**Fertilization methods**				0.059
**IVF**	2237(76.26%)	342(81.43%)	130(77.84%)	
**ICSI**	697(23.74%)	78(18.57%)	37(22.16%)	
**HCG day hormone levels**				
**LH (mIU/mL)**	1.12 ± 1.28^a^	1.12 ± 1.44^b^	0.80 ± 0.77^ab^	0.008
**E_2_ (pg/ml)**	3147.15 ± 1544.05^a^	4094.31 ± 1659.51^a^	4384.03 ± 1960.50^a^	<0.001
**P (ng/ml)**	0.78 ± 0.36^a^	1.89 ± 0.31^a^	2.18 ± 0.37^a^	<0.001
**Clinical pregnancy rate**	1979(67.24%)^a^	235(55.95%)^a^	76(45.51%)^a^	<0.001
**Abortion rate**	176(8.89%)	23(9.79%)	7(9.21%)	0.624

a, b Post-hoc pairwise comparisons P-value <0.05 for values with the same superscript letter; continuous variables are presented as mean ± SD; LFEP, late follicular elevated progesterone; BMI, body mass index; FSH, follicle-stimulating hormone; Gn, gonadotropin; ICSI, intracytoplasmic sperm injection; HCG, human chorionic gonadotropin; LH, luteinizing hormone; E_2_, estradiol; P, progesterone.

In the crude logistic regression analysis model shown in **Table 3**, clinical pregnancy-related factors were age, duration of infertility, Gn dosage, number of oocytes retrieved, number of embryos transferred, fertilized rate, LH level, and P level on the day of hCG administration. Irrespective of the definition of LFEP, the duration of LFEP was an influencing factor of clinical pregnancy rate when the reference was LFEP for 1 day. The crude odds ratio (ORs) for LFEP duration (≥ 2 days) were 0.753 (*P* = 0.008; LFEP defined as P > 1.0 ng/ml) and 0.657 (*P* = 0.023; LFEP defined as P > 1.5 ng/ml).

**Table 3:** Crude odds ratio for factors with clinical pregnancy rate.

	COR (95% CI)	*p-value*
**Age (y)**	0.912 (0.899-0.926)	<0.001
**Duration of infertility (y)**	0.936 (0.914-0.958)	<0.001
**BMI (Kg/m^2^)**	1.000 (0.978-1.022)	0.965
**Basic FSH (mIU/mL)**	0.971 (0.934-1.010)	0.145
**Gn duration (days)**	0.975 (0.943-1.007)	0.127
**Gn dosage (IU)**	1.000 (1.000-1.000)	<0.001
**Oocytes retrieved**	1.027 (1.013-1.040)	<0.001
**No. embryos transferred**	1.231 (0.861-1.304)	0.041
**Fertilization methods (IVF/ICSI)**	1.276 (1.078-1.510)	0.005
**HCG day hormone levels**		
**LH (mIU/mL)**	1.088 (1.027-1.154)	0.004
**E_2_ (pg/ml)**	1.000 (1.000-1.000)	0.356
**P (pg/ml)**	0.655 (0.581-0.740)	<0.001
**Duration of LFEP (1.0ng/ml)**		
**0 day (no LFEP)**	1.293 (1.089-1.536)	0.003
**1 day**	Reference	
**≥ 2 days**	0.753 (0.611-0.528)	0.008
**Duration of LFEP (1.5ng/ml)**		
**0 day (no LFEP)**	1.631 (1.326-2.008)	<0.001
**1 day**	Reference	
**≥ 2 days**	0.657(0.459-0.943)	0.023

COR, crude odds ratio; CI, confidence interval; BMI, body mass index; FSH, follicle-stimulating hormone; Gn, gonadotropin; LFEP, late follicular elevated Progesterone; HCG, human chorionic gonadotropin; LH, luteinizing hormone; E_2_, estradiol; P, progesterone.

In **Table 4**, multivariable logistic regression analysis was used to adjusting relative factors including age, duration of infertility, Gn dosage, oocytes retrieved, number of embryos transferred, and fertilized methods. When LFEP was defined as P > 1.0 ng/ml, LFEP was an independent risk factor for clinical pregnancy (adjusted OR = 1.290; *P* = 0.008). However, LFEP duration (≥ 2 days) was not associated with IVF outcome (adjusted OR = 0.808; *P* = 0.064). When LFEP was defined as P > 1.5 ng/ml, LFEP duration (≥ 2 days) was still not a predictive factor (adjusted OR = 0.720; *P* = 0.098).

**Table 4:** Adjusted odds ratio for factors with clinical pregnancy rate after multivariable logistic regression analysis.

	Duration of LFEP > 1.00 ng/mL		Duration of LFEP > 1.50 ng/mL	
	AOR (95% CI)	*p-value*	AOR (95% CI)	*p-value*
**Age (y)**	0.935(0.918-0.953)	<0.001	0.935(0.917-0.952)	<0.001
**Embryos transferred**	1.288(1.016-1.632)	0.036	1.300(1.027-1.645)	0.029
**Duration of LFEP**				
**0 day (no LFEP)**	1.290(1.070-1.555)	0.008	1.553(1.241-1.943)	<0.001
**1 day**	Reference		Reference	
**≥ 2 days**	0.808(0.644-1.013)	0.064	0.720(0.488-1.063)	0.098

Adjusted factors were age, duration of infertility, Gn dosage, oocytes retrieved, number of embryos transferred, fertilized methods, and duration of LFEP.

LFEP, late follicular elevated Progesterone; AOR, adjusted odds ratio; CI, confidence interval; Gn, gonadotropin.

## Discussion

Many studies have explored the impact of LFEP on IVF outcomes. There is nearly a consensus that LFEP impairs pregnancy rates in fresh embryo transfer cycles, while the definition of LFEP is still in debate. However, in our daily work, it is common to see that LFEP lasts for more than 1 day before hCG administration. Whether the duration of LFEP has an impact on IVF outcome is still not known. The current study showed that LFEP itself indeed was associated with a decreased clinical pregnancy rate. The duration of LFEP did not influence the clinical pregnancy rate in fresh embryo transfer cycles.

It has been proposed that elevated follicular P can alter the window of implantation, and therefore the transfer of an embryo in an asynchronous endometrium results in the failure to establishing embryo-endometrium cross-dialogue, which leads to embryonic demise and failure of implantation. Huang et al. proposed that the implantation window ranged from post-ovulatory day 6 to day 10; evaluating only one day of absolute serum P concentration might not accurately reflect the chronological change in the implantation window. Therefore, they analyzed the association between the duration of pre-ovulatory serum P elevation and the pregnancy outcomes of IVF/ICSI embryo transfer cycles (11). Results from that study showed that the duration of the premature serum P elevation was inversely related to the clinical pregnancy rate of IVF/ICSI-ET cycles. The endometrial receptivity analysis (ERA) was the first transcriptomic test developed to diagnose the endometrial receptivity status of infertile patients. ERA has never been presented independently of progesterone levels. The route of the activation of the progesterone receptor (PR) is not in debate. PR (A and B) activation is the main driver of the molecular changes that determine the WOI (window of implantation) and the initiation of pregnancy. It suggested that we can study the influence of LFEP duration on the WOI through the PR mechanism (13).

In the current study, LFEP duration was firstly shown to be associated with clinical pregnancy rate. It was interesting to see that LFEP duration was not a predictive factor for IVF outcome after adjusting for related parameters, and these results were inconsistent with another study (14). At first, we thought that 1.0 ng/ml was low and not enough to screen LFEP patients. However, the results were still the same when LFEP was defined as P > 1.5 ng/ml. LFEP was not a dependent incidence. The administration of relatively high doses of exogenous FSH for multi-follicular growth may contribute to the premature progesterone elevation (15). Recent evidence suggests FSH actively promotes granulosa cells to synthesis and output progesterone by upregulating the expression and increasing enzymatic activity of 3β-hydroxysterioddehydrogenoase (3β-HSD) without luteinization (16). In addition, previous data also showed that progesterone in the late follicular phase was also correlated with ovarian stimulation protocols, female age, and number of oocytes retrieved (17). These could explain the different effects of LFEP duration on IVF outcomes in crude and adjusted logistic regression analysis.

Then, why were the IVF outcomes different between patients with LFEP and those without LFEP but comparable in patients with LFEP for 1 day and ≥ 2 days? We speculate that the possible reason is that the number of progesterone receptors in the endometrium is certain. When progesterone in the late follicle phase reaches a certain level, the endometrium will change to the secretory stage in advance. However, with the increase of LFEP duration, the status of the endometrium no longer dramatically changes. Therefore, the effect of LFEP on IVF outcome for ≥ 2 days is the same as that for 1 day.

Our findings have several clinical implications. First, there has been robust evidence that LFEP impairs endometrial receptivity, but LFEP in the fresh cycle does not affect the cumulative birth rate of the frozen transfers in a freeze-only approach (18). A freeze-all policy should be proposed for LFEP patients to avoid the adverse impact of LFEP (19–21). We agree with the conclusion that LFEP has deleterious impact on fresh embryo transfers, but we should also be cautious regarding the cost of a freeze-all policy, especially for good-prognosis patients with sufficient high-quality embryos. Our data suggest that when we encounter such patients with ≥ 2days of LFEP, fresh embryo transfer should be cancelled. In addition, we should also be caution that our data do not suggest cancelling continuous monitoring of progesterone before hCG administration, as progesterone level is also indictor for oocyte maturation and hCG administration (22, 23).

A strength of the current larger population-based study was that all patients were derived from a single-center study, and were all treated with pituitary downregulation protocols. In addition, the effect of LFEP duration on IVF outcomes was explored using two different LFEP criteria. However, several limitations also existed. Not all factors could be controlled due to the retrospective nature of this study. The specific mechanism for this phenomenon still needs to be explored in further basic experiments. Moreover, the impact of LFEP duration and cumulative pregnancy outcomes with transferred frozen embryos should also be demonstrated in the future.

## Conclusion

Taken altogether, our data showed that irrespective of LFEP criteria (i.e., whether LFEP was defined as P > 1.0 ng/ml or as P > 1.5 ng/ml), LFEP adversely affects clinical pregnancy outcomes. However, compared with a duration of LFEP of 1 day, the longer duration of LFEP ≥ 2 days seems to have no influence on the clinical pregnancy rate in pituitary downregulation treatment cycles.

## Data availability statement

The original contributions presented in the study are included in the article/supplementary material. Further inquiries can be directed to the corresponding author.

## Ethics statement

The studies involving human participants were reviewed and approved by Zhengzhou University Research Ethics Board. The patients/participants provided their written informed consent to participate in this study.

## Author contributions

JZ contributed to the study design, data analysis, and manuscript preparation. XG handled patient recruitment and data collection. ZB supervised this study. All authors read and approved the final manuscript.

## References

[1] Roque M Valle M Sampaio M Geber S Checa MA. Ratio of progesterone-to-number of follicles as a prognostic tool for *in vitro* fertilization cycles. J Assist Reprod Genet (2015) 32:951–7. doi: 10.1007/s10815-015-0487-1 PMC4491074 25925350

[2] Martinez F Coroleu B Clua E Tur R Buxaderas R Parera N. Serum progesterone concentrations on the day of HCG administration cannot predict pregnancy in assisted reproduction cycles. Reprod BioMed Online (2004) 8:183–90. doi: 10.1016/S1472-6483(10)60514-7 14989795

[3] Venetis CA Kolibianakis EM Papanikolaou E Bontis J Devroey P Tarlatzis BC. Is progesterone elevation on the day of human chorionic gonadotrophin administration associated with the probability of pregnancy in *in vitro* fertilization? a systematic review and meta-analysis. Hum Reprod Update (2007) 13:343–55. doi: 10.1093/humupd/dmm007 17405832

[4] Lawrenz B Labarta E Fatemi H Bosch E. Premature progesterone elevation: targets and rescue strategies. FERTIL STERIL (2018) 109:577–82. doi: 10.1016/j.fertnstert.2018.02.128 29653703

[5] Yding AC Bungum L Nyboe AA Humaidan P. Preovulatory progesterone concentration associates significantly to follicle number and LH concentration but not to pregnancy rate. Reprod BioMed Online (2011) 23:187–95. doi: 10.1016/j.rbmo.2011.04.003 21665546

[6] Venetis CA Kolibianakis EM Bosdou JK Tarlatzis BC. Progesterone elevation and probability of pregnancy after IVF: a systematic review and meta-analysis of over 60 000 cycles. Hum Reprod Update (2013) 19:433–57. doi: 10.1093/humupd/dmt014 23827986

[7] Wu Z Li R Ma Y Deng B Zhang X Meng Y. Effect of HCG-day serum progesterone and oestradiol concentrations on pregnancy outcomes in GnRH agonist cycles. Reprod BioMed Online (2012) 24:511–20. doi: 10.1016/j.rbmo.2012.02.003 22417667

[8] Shufaro Y Sapir O Oron G HA B Garor R Pinkas H. Progesterone-to-follicle index is better correlated with *in vitro* fertilization cycle outcome than blood progesterone level. FERTIL STERIL (2015) 103:669–74. doi: 10.1016/j.fertnstert.2014.11.026 25544249

[9] Hill MJ Healy MW Richter KS Widra E Levens ED De Cherney AH. Revisiting the progesterone to oocyte ratio. FERTIL STERIL (2017) 107:671–6. doi: 10.1016/j.fertnstert.2016.11.019 PMC5337440 28069176

[10] Zhang Y Xu Y Wang Y Xue Q Shang J Yang X. Comparison of the predictive value of progesterone-related indicators for pregnancy outcomes of women undergoing the short-acting GnRH agonist long protocol: a retrospective study. J Ovarian Res (2021) 14:14. doi: 10.1186/s13048-021-00768-2 33436055 PMC7802138

[11] Huang CC Lien YR Chen HF Chen MJ Shieh CJ Yao YL. The duration of pre-ovulatory serum progesterone elevation before hCG administration affects the outcome of IVF/ICSI cycles. Hum Reprod (2012) 27:2036–45. doi: 10.1093/humrep/des141 22561057

[12] Santos-Ribeiro S Racca A Roelens C De Munck N Mackens S Drakopoulos P. Evaluating the benefit of measuring serum progesterone prior to the administration of HCG: effect of the duration of late-follicular elevated progesterone following ovarian stimulation on fresh embryo transfer live birth rates. Reprod BioMed Online (2019) 38:647–54. doi: 10.1016/j.rbmo.2018.11.016 30593439

[13] Ruiz-Alonso M Valbuena D Gomez C Cuzzi J Simon C. Endometrial receptivity analysis (ERA): data versus opinions. Hum Reprod (2021) 2:1–11. doi: 10.1093/hropen/hoab011 PMC8045472 33880420

[14] Dai W Bu ZQ Wang LL Sun YP. The relationship between the changes in the level of progesterone and the outcome of *in vitro* fertilization-embryo transfer. Syst Biol Reprod Med (2015) 61:388–97. doi: 10.3109/19396368.2015.1064489 26247832

[15] Kyrou D Kolibianakis EM Fatemi HM Tarlatzis BC Tournaye H Devroey P. Is earlier administration of human chorionic gonadotropin (hCG) associated with the probability of pregnancy in cycles stimulated with recombinant follicle-stimulating hormone and gonadotropin-releasing hormone (GnRH) antagonists? a prospective randomized trial. FERTIL STERIL (2011) 96:1112–5. doi: 10.1016/j.fertnstert.2011.08.029 21924414

[16] Oktem O Akin N Bildik G Yakin K Alper E Balaban B. FSH stimulation promotes progesterone synthesis and output from human granulosa cells without luteinization. Hum Reprod (2017) 32:643–52. doi: 10.1093/humrep/dex010 28158500

[17] Bu Z Zhao F Wang K Guo Y Su Y Zhai J. Serum progesterone elevation adversely affects cumulative live birth rate in different ovarian responders during *in vitro* fertilization and embryo transfer: a large retrospective study. PloS One (2014) 9:e100011. doi: 10.1371/journal.pone.0100011 24926883 PMC4057273

[18] Racca A Vanni VS Somigliana E Reschini M Vigano P Santos-Ribeiro S. Is a freeze-all policy the optimal solution to circumvent the effect of late follicular elevated progesterone? a multicentric matched-control retrospective study analysing cumulative live birth rate in 942 non-elective freeze-all cycles. Hum Reprod (2021) 36:2463–72. doi: 10.1093/humrep/deab160 34223890

[19] Roque M. Freeze-all policy: is it time for that? J Assist Reprod Genet (2015) 32:171–6. doi: 10.1007/s10815-014-0391-0 PMC4354191 25428436

[20] Xu B Li Z Zhang H Jin L Li Y Ai J. Serum progesterone level effects on the outcome of *in vitro* fertilization in patients with different ovarian response: an analysis of more than 10,000 cycles. FERTIL STERIL (2012) 97:1321–7. doi: 10.1016/j.fertnstert.2012.03.014 22494924

[21] Merviel P Bouee S Jacamon AS Chabaud JJ Le Martelot MT Roche S. Progesterone levels on the human chorionic gonadotropin trigger day affect the pregnancy rates for embryos transferred at different stages of development in both general and selected IVF/ICSI populations. BMC Pregnancy Childbirth (2021) 21:363. doi: 10.1186/s12884-021-03832-3 33957886 PMC8101180

[22] Reynaud K Saint-Dizier M Tahir MZ Havard T Harichaux G Labas V. Progesterone plays a critical role in canine oocyte maturation and fertilization. Biol Reprod (2015) 93:87. doi: 10.1095/biolreprod.115.130955 26333993

[23] Turgut EN Ecemis S Boynukalin KF Gultomruk M Yarkiner Z Findikli N. Being on the side of old findings: progesterone elevation on the day of oocyte maturation induction does not affect embryological parameters throughout the blastocyst culture period. Arch GYNECOL OBSTET (2021) 303:581–7. doi: 10.1007/s00404-020-05792-z 32918591

